# Supplementary material for: Directional Anisotropy of the Vibrational Modes in 2D-Layered Perovskites
Source: ACS Nano. 2020 Apr 10;14(4):4689–97. doi: 10.1021/acsnano.0c00435 (PMC8007126; doi:10.1021/acsnano.0c00435)
Supplement: Supplementary file 1 — nn0c00435_si_001.pdf [file nn0c00435_si_001.pdf]

SUPPORTING INFORMATION

Directional Anisotropy of the Vibrational Modes in 2D Layered Perovskites

*Balaji Dhanabalan[1,2] ‡, Yu-Chen Leng[3,4] ‡, Giulia Biffi[1,2], Miao-Ling Lin[3,4], Ping-Heng Tan[3,4], Ivan Infante[1], Liberato Manna[1], Milena P. Arciniegas[1], and Roman Krahne[1]**

[1]*Istituto Italiano di Tecnologia (IIT), Via Morego 30, 16163 Genoa, Italy*

[2]*Dipartimento di Chimica e Chimica Industriale, Università degli Studi di Genova, Via Dodecaneso, 31, 16146 Genova, Italy*

[3]*State Key Laboratory of Superlattices and Microstructures, Institute of Semiconductors, Chinese Academy of Sciences, 100083 Beijing, China*

[4]*Center of Materials Science and Optoelectronics Engineering, University of Chinese Academy of Sciences, 100190 Beijing, China*

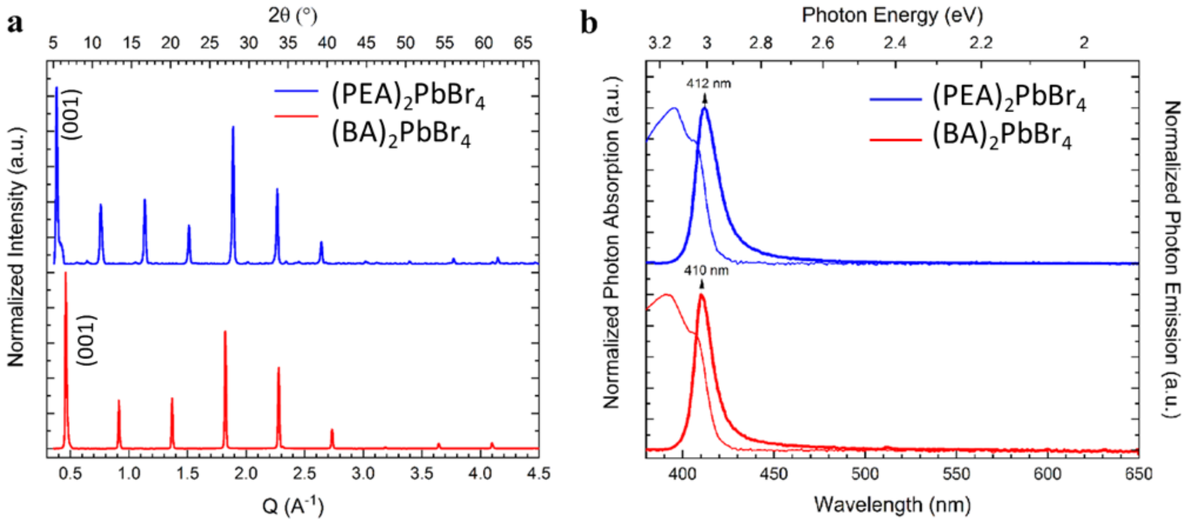

Figure S1 – (a) X-Ray Diffraction pattern collected from $(PEA)_2PbBr_4$ and $(BA)_2PbBr_4$ flakes showing the periodical peaks with inter-distance of 16.68 ± 0.07 Å and 13.83 ± 0.06 Å, respectively. The inter-distance corresponds to the stacking of inorganic $[PbBr4]^{2-}$ octahedral layers separated by organic molecules, confirming the *n*=1 structure. The equally spaced reflections observed, following the {00l} family, indicate the dominant orientation of the flakes with their basal plane parallel to the substrate; that is the octahedral layers are parallel to the substrate. (b) Absorbance and emission of exfoliated flakes showing the photoluminescence peak at 412 nm for $(PEA)_2PbBr_4$ and at 410 nm for $(BA)_2PbBr_4$.

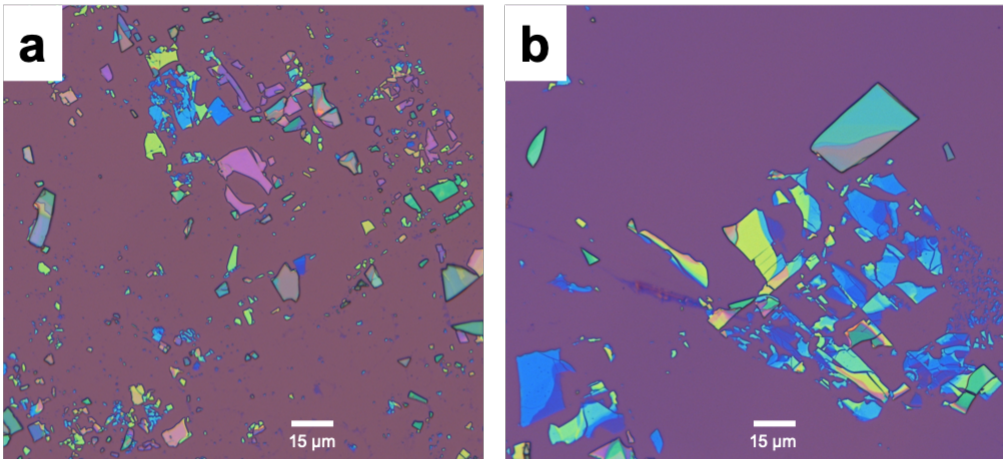

Figure S2 – Images evidencing the frequent occurrence of straight edges and rectangular shapes of the exfoliated $(PEA)_2PbBr_4$ (a) and $(BA)_2PbBr_4$ (b) flakes.

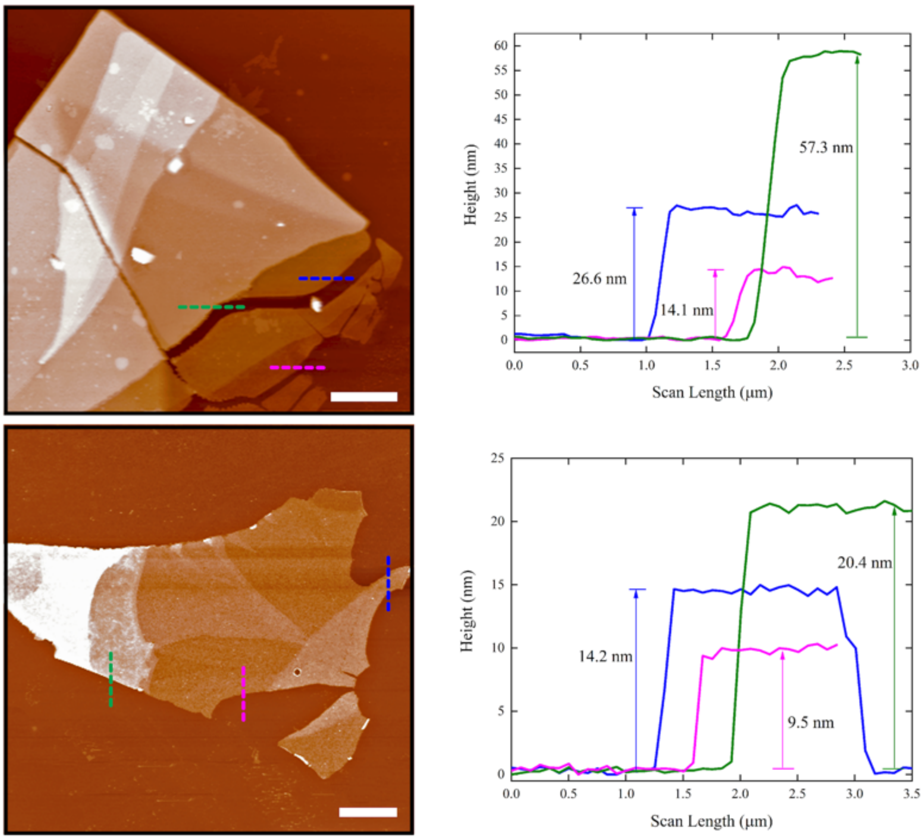

Figure S3 – Atomic force microscopy (AFM) images showing the topography of representative $(PEA)_2PbBr_4$ (top) and $(BA)_2PbBr_4$ (bottom) flakes. The number of organic/inorganic bilayers can be obtained from the average height divided by the interlayer distance, which is 1.4 nm for $(BA)_2PbBr_4$, and 1.7 nm for $(PEA)_2$PbBr4 flakes, *i.e.* approximately 8, 16, and 34 layers for $(PEA)_2PbBr_4$, and 7, 10 and 14 layers for $(BA)_2PbBr_4$. The scale bars correspond to 5 μm.

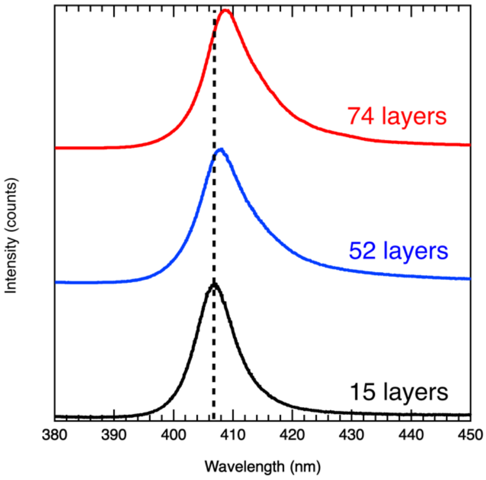

Figure S4 – Photoluminescence (PL) spectra recorded from single $(PEA)_2PbBr_4$ flakes with different thickness. The dashed line is a guide to the eye, highlighting the red-shift of the peak maximum to longer wavelengths with increasing layer number. This small shift in PL peak position can be tentatively attributed to self-absorption.

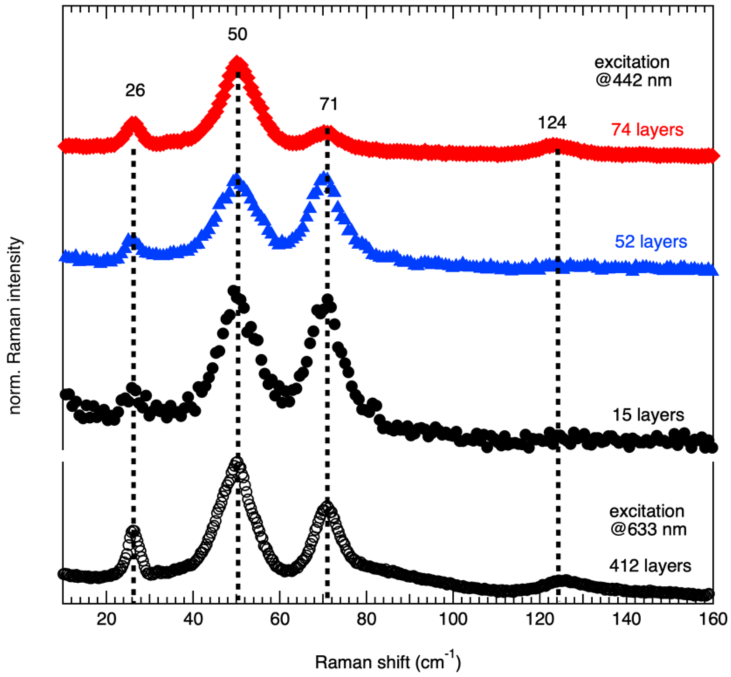

Figure S5 – Raman spectra recorded with laser excitation at 442 nm from $(PEA)_2PbBr_4$ flakes with different thicknesses (full symbols), and with 633 nm laser excitation from a flake with 412 octahedral layers. The position of the observed Raman peaks does not depend significantly on the laser excitation wavelength or number of layers. Weak Raman signals can be better discerned under excitation with 633 nm light, because at this wavelength much below the band edge of the 2D flake the emission is weak and therefore the background is reduced.

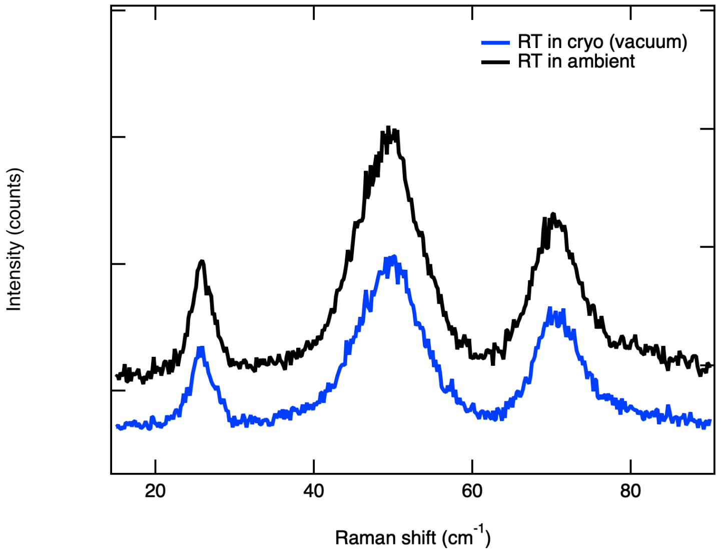

Figure S6 – Raman spectra recorded from a $(PEA)_2PbBr_4$ flake under vacuum and in ambient air at room temperature, indicating that the different environments have no significant impact on the Raman scattering. The excitation wavelength was 633 nm.

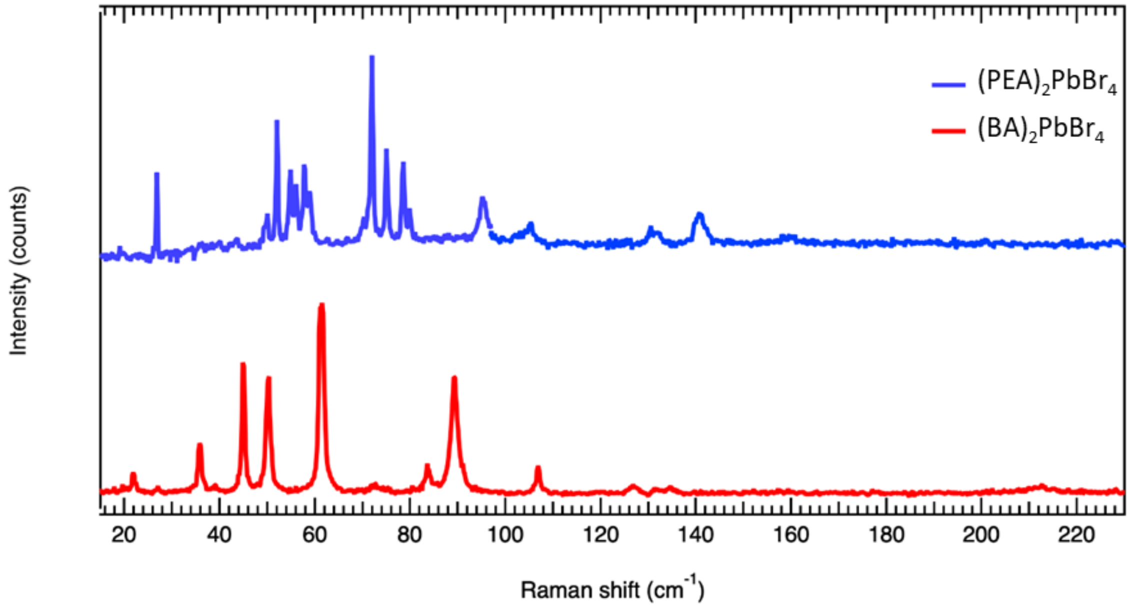

Figure S7 – Ultralow-frequency Raman spectra of individual $(PEA)_2PbBr_4$ and $(BA)_2PbBr_4$ perovskite flakes recorded at T=4 K under laser excitation at 633 nm.

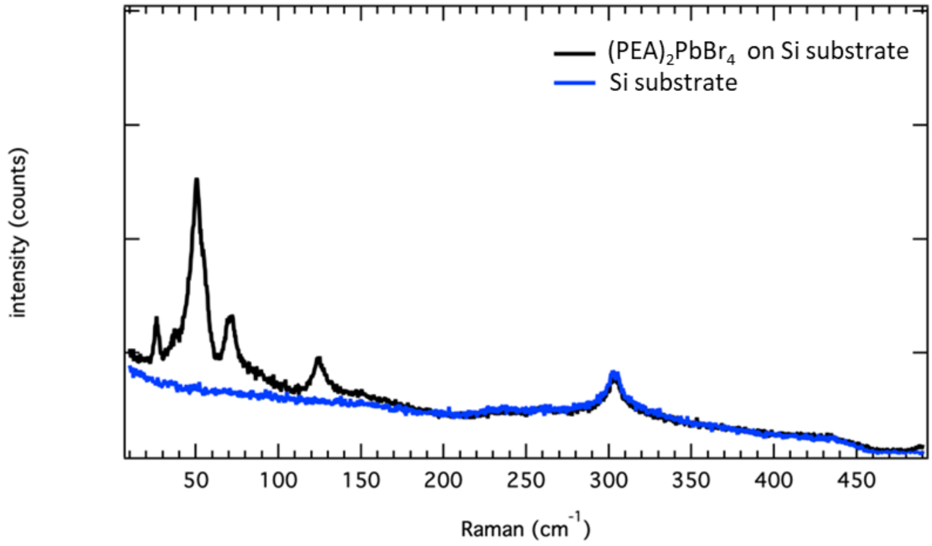

Figure S8 – Raman spectra recorded with 633 nm excitation from a $(PEA)_2PbBr_4$ flake and from the underlying Si substrate.

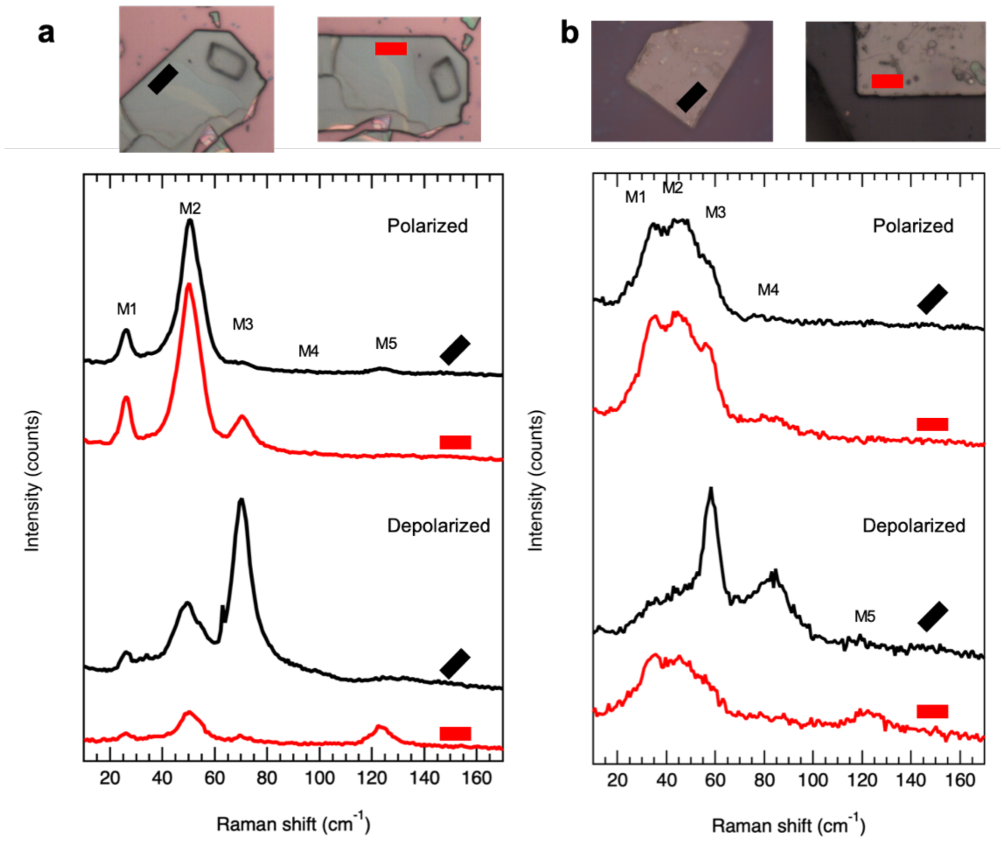

Figure S9 – Polarization-dependent Raman spectra of $(PEA)_2PbBr_4$ and $(BA)_2PbBr_4$ flakes for different orientations recorded at room temperature. The microscope images on the top show the flake orientation.

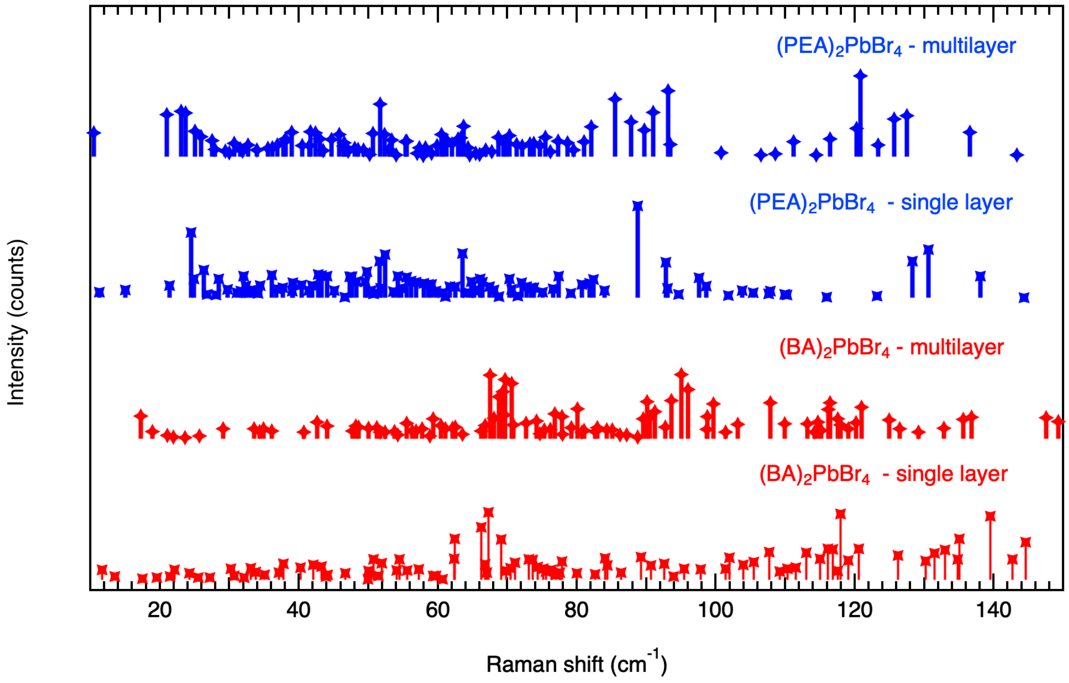

Figure S10 – Normal mode analysis by density functional theory (DFT) calculations for multi- and single-layer $(PEA)_2PbBr_4$ and $(BA)_2PbBr_4$ structures. See also movie animations in the Supporting Information.

Raman intensities derived from group theory analysis.

According to the assignments in Table 1, we derive the polarization of the M1-M5 bands as follows. The Raman tensors of the A_g, B_{1g}, B_{2g} and B_{3g} symmetries of the D_{2h} group are presented by:

$$A_g: \begin{pmatrix} a & 0 & 0 \\ 0 & b & 0 \\ 0 & 0 & c \end{pmatrix}$$

$$B_{1g}: \begin{pmatrix} 0 & d & 0 \\ d & 0 & 0 \\ 0 & 0 & 0 \end{pmatrix}$$

$$B_{2g}: \begin{pmatrix} 0 & 0 & e \\ 0 & b & 0 \\ e & 0 & 0 \end{pmatrix}$$

$$B_{3g}: \begin{pmatrix} 0 & 0 & 0 \\ 0 & 0 & f \\ 0 & f & 0 \end{pmatrix}$$

The intensity of a Raman-active mode is expressed by the Raman tensor (R_j) as:

$$\sum_j \left| e_s \cdot R_j \cdot e_i \right|^2$$

For polarization parallel to the axes of the pseudocubic octahedral lattice, e_s and e_i are presented by:

Polarized: $e_s = e_i = (1,0,0)$

It follows that only the intensity of A_g (thus for M2, M4 and M5) is not 0.

Depolarized: $e_s = (1,0,0)$; $e_i = (0,1,0)$

It follows that only the intensity of B_{1g} (M1) is not 0.

For polarization at 45° to the main axes, e_s and e_i are presented as:

Polarized: $e_s = e_i = (\frac{\sqrt{2}}{2}, \frac{\sqrt{2}}{2}, 0)$

It follows that only the intensity of A_g (thus for M2, M4 and M5) and B_{1g} (M1) are not 0, with $I(A_g) \propto \frac{1}{4}(a+b)^2$. Therefore, M2, M4 and M5 are present in polarized Raman spectra.

Depolarized: $e_s = (\frac{\sqrt{2}}{2}, \frac{\sqrt{2}}{2}, 0)$; $e_i = (\frac{\sqrt{2}}{2}, -\frac{\sqrt{2}}{2}, 0)$

Only the intensity of A_g (M2, M4 and M5) is not 0, with $I(A_g) \propto \frac{1}{4}(a-b)^2$. In this case, if a=b, all the modes should disappear. That is, A_g mode may be observed in this case, depending on the values of a and b.
