# Supplementary figures and images for: Directional Anisotropy of the Vibrational Modes in 2D-Layered Perovskites
Source: ACS Nano. 2020 Apr 10;14(4):4689–97. doi: 10.1021/acsnano.0c00435 (PMC8007126; doi:10.1021/acsnano.0c00435)

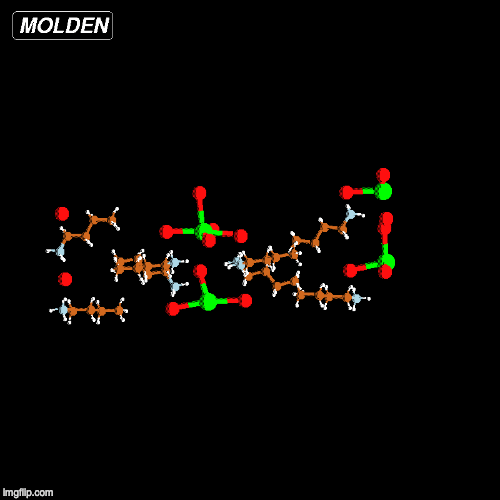

Supplement: Supplementary file 2 — nn0c00435_si_002.zip [file nn0c00435_si_002.zip › BA_43cm-1.gif]

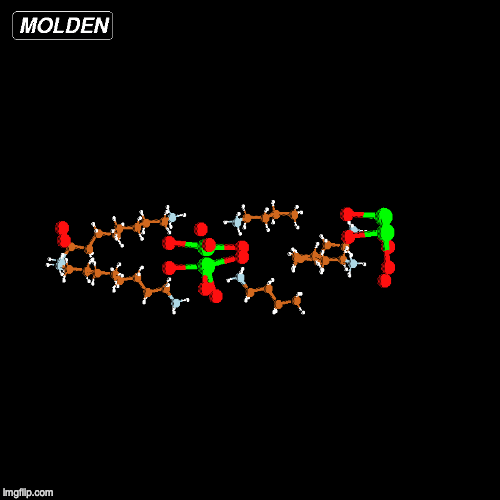

Supplement: Supplementary file 2 — nn0c00435_si_002.zip [file nn0c00435_si_002.zip › BA_58cm-1.gif]

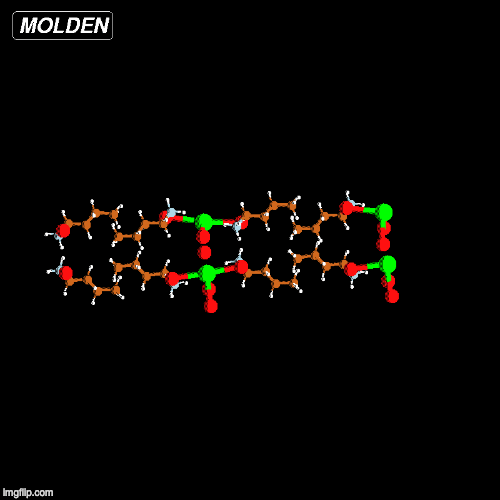

Supplement: Supplementary file 2 — nn0c00435_si_002.zip [file nn0c00435_si_002.zip › BA_92cm-1.gif]

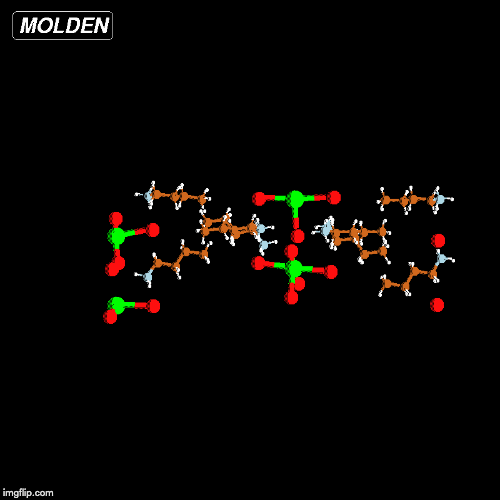

Supplement: Supplementary file 2 — nn0c00435_si_002.zip [file nn0c00435_si_002.zip › BA_137_cm-1.gif]

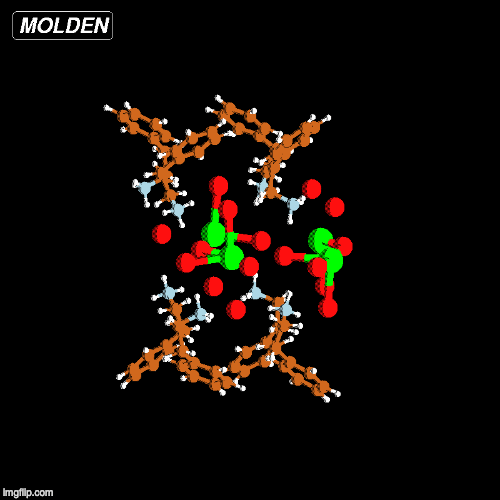

Supplement: Supplementary file 2 — nn0c00435_si_002.zip [file nn0c00435_si_002.zip › PEA_43cm-1.gif]

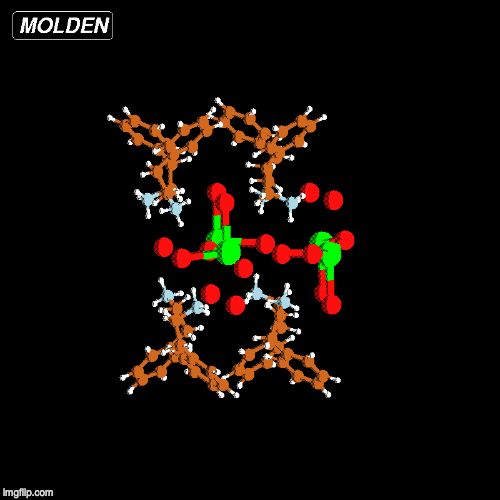

Supplement: Supplementary file 2 — nn0c00435_si_002.zip [file nn0c00435_si_002.zip › PEA_59cm-1.gif]

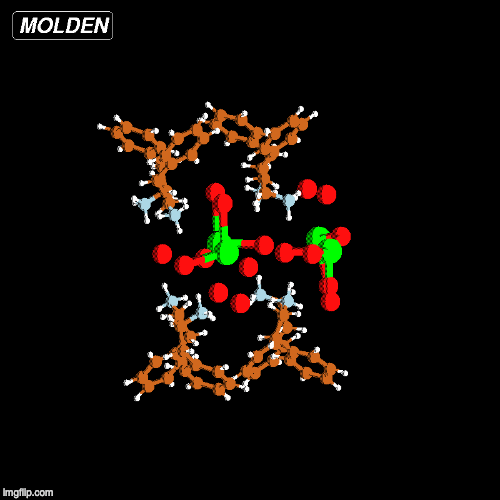

Supplement: Supplementary file 2 — nn0c00435_si_002.zip [file nn0c00435_si_002.zip › PEA_76_cm-1.gif]

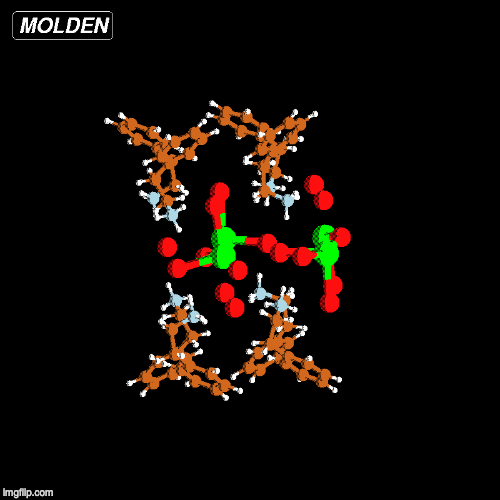

Supplement: Supplementary file 2 — nn0c00435_si_002.zip [file nn0c00435_si_002.zip › PEA_100cm-1.gif]

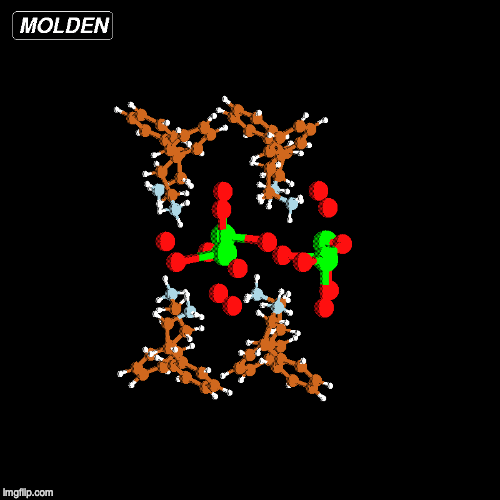

Supplement: Supplementary file 2 — nn0c00435_si_002.zip [file nn0c00435_si_002.zip › PEA_135cm-1.gif]
